# Supplementary figures and images for: Impact of cerebrospinal fluid leukocyte infiltration and activated neuroimmune mediators on survival with HIV-associated cryptococcal meningitis
Source: PLoS Negl Trop Dis. 2025 Feb 10;19(2):e0012873. doi: 10.1371/journal.pntd.0012873 (PMC11844869; doi:10.1371/journal.pntd.0012873)

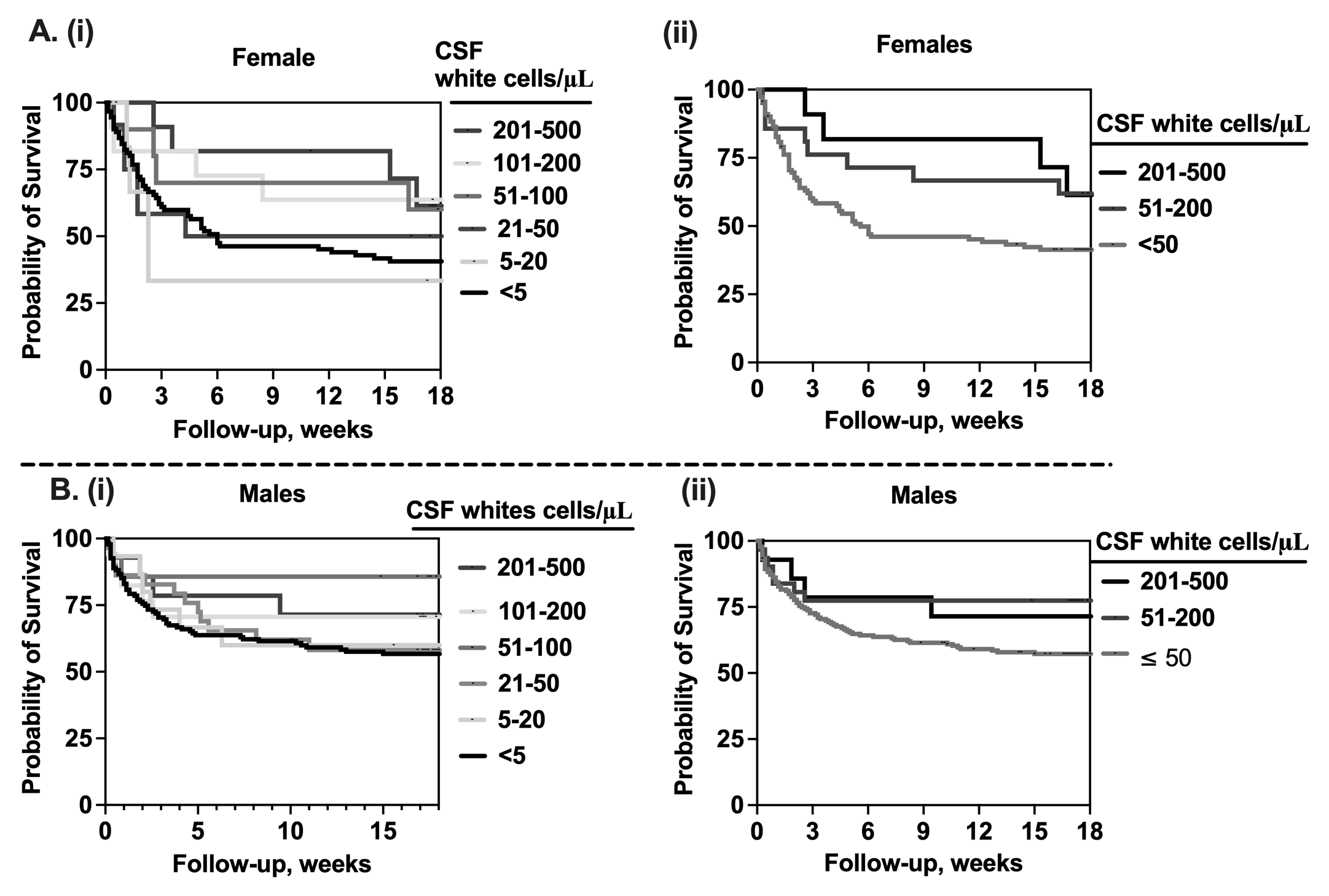

Supplement: S1 Fig — Correlation of CSF cytokines and chemokine levels with CSF leukocyte counts (≤50 cells/μL and >50 cells/μL by survival. A cryptococcal CSF fungal burden–log 10 CFU–colony forming units- Interleukin 2, A (ii)—Interferon gamma, A (iii)—Tumor necrosis factor alpha. B—CXCL10/IP-10. C—CCL11/Eotaxin. D–interferon gamma. E—Th17 cytokine, IL-17A. The interlinking bars–shows two variable unpaired comparison. Error bars–show median and 95% CI. Asterisks *—show statistically significant variables reported at p-value <0.050, at 95% confidence intervals. (TIF) [file pntd.0012873.s001.tif]

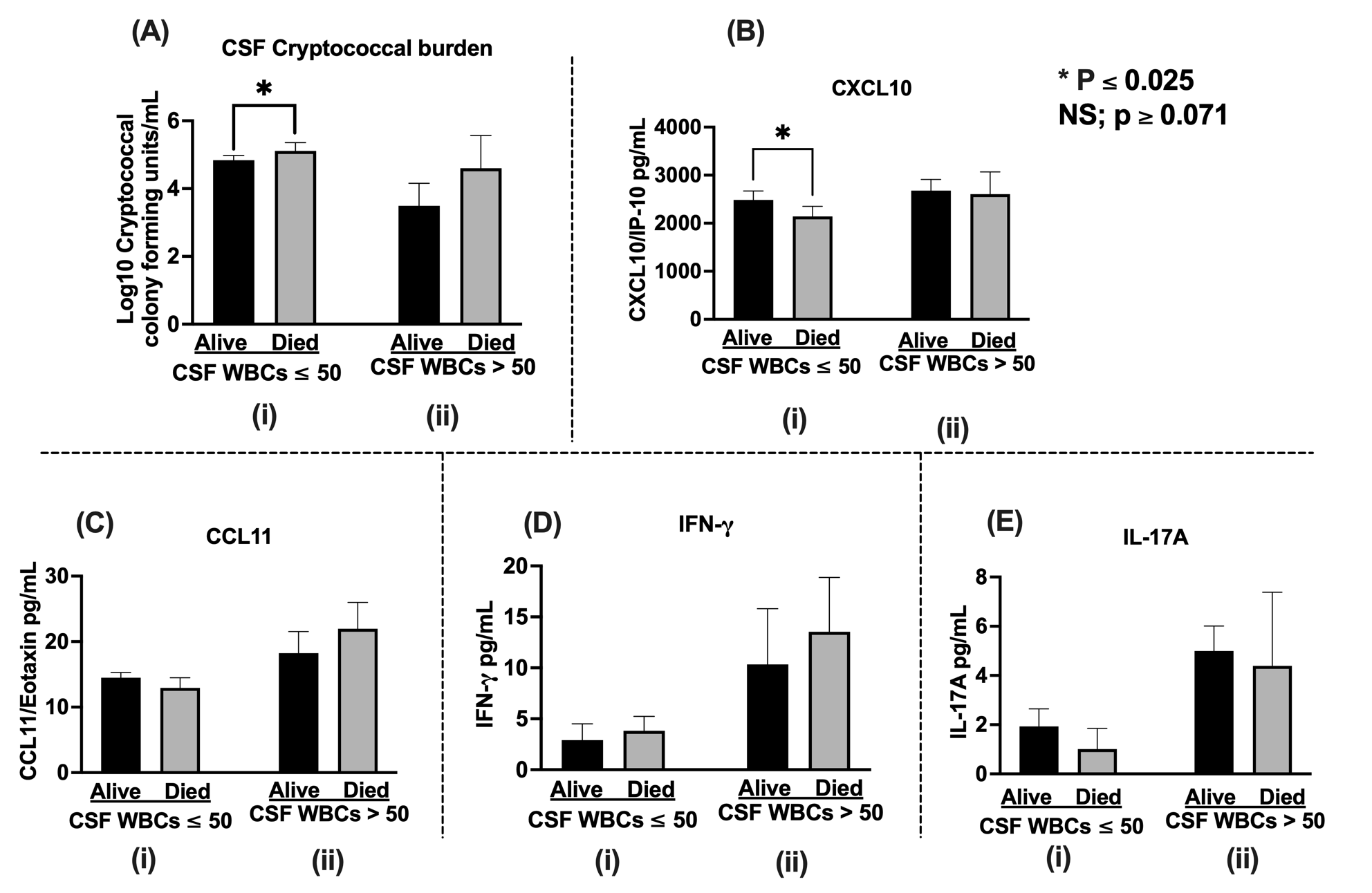

Supplement: S2 Fig — (TIF) [file pntd.0012873.s002.tif]
