# Supplementary material for: Impact of cerebrospinal fluid leukocyte infiltration and activated neuroimmune mediators on survival with HIV-associated cryptococcal meningitis
Source: PLoS Negl Trop Dis. 2025 Feb 10;19(2):e0012873. doi: 10.1371/journal.pntd.0012873 (PMC11844869; doi:10.1371/journal.pntd.0012873)
Supplement: S1 Table — (DOCX) [file pntd.0012873.s003.docx]

**Supplementary S1 Table. Multiple adjusted differences in the peripheral and Cerebrospinal Fluid Clinical Variables by Set Cerebrospinal Fluid Leukocyte Count.**

| Set Cerebrospinal fluid Leukocytes /Microliter or Cytokine or Chemokine | Class Median (Interquartile Range) | Multiple Benjamini,  Krieger and Yekutieli  Adjusted Classes | Adjusted Mean  Rank Differences | Individual  p Values | Adjusted q Values |
| --- | --- | --- | --- | --- | --- |
| CD4 T cells, /µL by set CSF Leukocytes | | | | | |
| <50 cells/ µL | 12.5 (6.0-33.3) | <50 vs 51-200 cells/ µL | -69.47 | <0.001 | <0.001 |
| 51-200 cells/ µL | 38.0 (10.0-76.0) | <50 vs. 201-500 cells/ µL | -100.6 | <0.001 | <0.001 |
| 201-500 cells/ µL | 57.0 (22.0-87.0) | 51-200 vs. 201-500 cells/ µL | -31.11 | 0.25 | 0.09 |
| CD8 T cells, /µL by Set CSF Leukocytes levels | | | | | |
| <50 cells/ µL | 273.5 (160.3-464.0) | <50 vs 51-200 cells/ µL | -58.77 | <0.001 | <0.001 |
| 51-200 cells/ µL | 418.0 (264.0-826.0) | <50 vs. 201-500 cells/ µL | -62.99 | 0.006 | 0.003 |
| 201-500 cells/ µL | 420.0 (203.5-1170.0) | 51-200 vs. 201-500 cells/ µL | -4.225 | 0.87 | 0.31 |
| Peripheral Leukocytes, x10^3^/µL by Set CSF Leukocytes | | | | | |
| <50 cells/ µL | 3.3 (2.5-5.2) | <50 vs 51-200 cells/ µL | -13.03 | 0.42 | 0.44 |
| 51-200 cells/ µL | 3.7 (3.0-4.4) | <50 vs. 201-500 cells/ µL | -46.56 | 0.05 | 0.16 |
| 201-500 cells/ µL | 3.9 (3.4-5.1) | 51-200 vs. 201-500 cells/ µL | -33.53 | 0.22 | 0.34 |
| Hemoglobin, g/dL by Set CSF Leukocytes | | | | | |
| <50 cells/ µL | 11.7 (10.1-13.3) | <50 vs 51-200 cells/ µL | -5.407 | 0.74 | 0.77 |
| 51-200 cells/ µL | 11.4 (10.1-13.6) | <50 vs. 201-500 cells/ µL | 46.45 | 0.05 | 0.09 |
| 201-500 cells/ µL | 10.5 (9.2-12.4) | 51-200 vs. 201-500 cells/ µL | 51.86 | 0.06 | 0.09 |
| CSF fungal QCC /mL by Set CSF Leukocytes | | | | | |
| <50 cells/ µL | 4.9 (3.7-5.6) | <50 vs 51-200 cells/ µL | 54.16 | 0.001 | <0.001 |
| 51-200 cells/ µL | 3.8 (2.2-4.9) | <50 vs. 201-500 cells/ µL | 93.18 | <0.001 | <0.001 |
| 201-500 cells/ µL | 2.3 (1.6-4.6) | 51-200 vs. 201-500 cells/ µL | 39.02 | 0.16 | 0.05 |
| CSF Proteins, mg/dL by set CSF Leukocytes | | | | | |
| <50 cells/ µL | 40.0 (22.0-80.5) | <50 vs 51-200 cells/ µL | -59.35 | <0.001 | <0.001 |
| 51-200 cells/ µL | 100.5 (29.8-160.0) | <50 vs. 201-500 cells/ µL | -108.6 | <0.001 | <0.001 |
| 201-500 cells/ µL | 143 (82.5-182.0) | 51-200 vs. 201-500 cells/ µL | -49.24 | 0.04 | 0.04 |
| CSF Opening Pressure, mmHg by set CSF Leukocytes | | | | | |
| <50 cells/ µL | 29.1 (20.0-44.0) | <50 vs 51-200 cells/ µL | 14.1 | 0.36 | 0.93 |
| 51-200 cells/ µL | 26.0 (18.0-41.3) | <50 vs. 201-500 cells/ µL | 0.7023 | 0.97 | >0.99 |
| 201-500 cells/ µL | 28.2 (20.8-39.3) | 51-200 vs. 201-500 cells/ µL | -13.4 | 0.59 | 0.93 |
| CSF Glucose, mg/dL by set CSF Leukocytes | | | | | |
| <50 cells/ µL | 72.0 (44.5-102.5) | <50 vs 51-200 cells/ µL | 22.86 | 0.05 | 0.17 |
| 51-200 cells/ µL | 31.5 (26.8-93.5) | <50 vs. 201-500 cells/ µL | 4.433 | 0.78 | 0.82 |
| 201-500 cells/ µL | 83.0 (37.0-101-0) | 51-200 vs. 201-500 cells/ µL | -18.43 | 0.34 | 0.53 |

Summary statistics from the two-stage linear step-up adjustment for multiple colinear differences in variable by set CSF leukocyte count using Benjamini, Krieger and Yekutieli model analysis. Unadjusted p Value <0.05 was consistently significant. The adjusted q value <0.05 was consistently significant.
