# Supplementary material for: Impact of cerebrospinal fluid leukocyte infiltration and activated neuroimmune mediators on survival with HIV-associated cryptococcal meningitis
Source: PLoS Negl Trop Dis. 2025 Feb 10;19(2):e0012873. doi: 10.1371/journal.pntd.0012873 (PMC11844869; doi:10.1371/journal.pntd.0012873)
Supplement: S2 Table — (DOCX) [file pntd.0012873.s004.docx]

**Supplementary S2 Table. Multiple adjusted differences in the Cerebrospinal Fluid Clinical Soluble Factors by Set Cerebrospinal Fluid Leukocyte Count.**

| Set Cerebrospinal Fluid Leukocytes /Microliter or Cytokine or Chemokine | Class Median (Interquartile Ranges) | Multiple Benjamini,  Krieger and Yekutieli  Adjusted Classes | Adjusted Mean Rank Differences | Individual  p Values | Adjusted q Values |
| --- | --- | --- | --- | --- | --- |
| IL-2 cytokine levels by set CSF Leukocytes levels | | | | | |
| <50 cells/ µL | 4.3 (2.1-7.8) | <50 vs 51-200 cells/ µL | -71.93 | <0.001 | <0.001 |
| 51-200 cells/ µL | 8.7 (3.8-11.9) | <50 vs. 201-500 cells/ µL | -97.12 | <0.001 | <0.001 |
| 201-500 cells/ µL | 8.8 (6.1-13.4) | 51-200 vs. 201-500 cells/ µL | -25.18 | 0.36 | 0.13 |
| IL-12p70 cytokine levels by set CSF Leukocytes levels | | | | | |
| <50 cells/ µL | 5.0 (1.0-9.2) | <50 vs 51-200 cells/ µL | -56.51 | <0.001 | <0.001 |
| 51-200 cells/ µL | 8.5 (3.3-13.7) | <50 vs. 201-500 cells/ µL | -53.89 | 0.02 | 0.01 |
| 201-500 cells/ µL | 7.2 (4.3-13.2) | 51-200 vs. 201-500 cells/ µL | 2.617 | 0.92 | 0.32 |
| TNF- α cytokine levels by set CSF Leukocytes levels | | | | | |
| <50 cells/ µL | 34.5 (12.8-84.4) | <50 vs 51-200 cells/ µL | -80.73 | <0.001 | <0.001 |
| 51-200 cells/ µL | 84.9 (43.7-148.6) | <50 vs. 201-500 cells/ µL | -96.85 | <0.001 | <0.001 |
| 201-500 cells/ µL | 89.4 (60.0-155.3) | 51-200 vs. 201-500 cells/ µL | -16.12 | 0.56 | 0.19 |
| IFN-γ cytokine levels by set CSF Leukocytes levels | | | | | |
| <50 cells/ µL | 2.9 (0.4-8.9) | <50 vs 51-200 cells/ µL | -76.41 | <0.001 | <0.001 |
| 51-200 cells/ µL | 11.2 (2.7-18.4) | <50 vs. 201-500 cells/ µL | -109.4 | <0.001 | <0.001 |
| 201-500 cells/ µL | 14.7 (7.3-21.7) | 51-200 vs. 201-500 cells/ µL | -33.01 | 0.23 | 0.08 |
| IL-17A cytokine levels by set CSF Leukocytes levels | | | | | |
| <50 cells/ µL | 1.2 (0.2-3.7) | <50 vs 51-200 cells/ µL | -85.82 | <0.001 | <0.001 |
| 51-200 cells/ µL | 4.4 (2.1-6.9) | <50 vs. 201-500 cells/ µL | -118.8 | <0.001 | <0.001 |
| 201-500 cells/ µL | 5.9 (3.6-15.3) | 51-200 vs. 201-500 cells/ µL | -32.96 | 0.23 | 0.08 |
| IL-4 cytokine levels by set CSF Leukocytes levels | | | | | |
| <50 cells/ µL | 1.2 (0.5-2.3) | <50 vs 51-200 cells/ µL | -20.52 | 0.22 | 0.57 |
| 51-200 cells/ µL | 1.7 (0.7-2.3) | <50 vs. 201-500 cells/ µL | -21.52 | 0.36 | 0.57 |
| 201-500 cells/ µL | 1.6 (0.7-2.1) | 51-200 vs. 201-500 cells/ µL | -1.001 | 0.97 | >0.99 |
| IL-6 cytokine levels by set CSF Leukocytes levels | | | | | |
| <50 cells/ µL | 124.8 (36.9-659.9) | <50 vs 51-200 cells/ µL | -72.94 | <0.001 | <0.001 |
| 51-200 cells/ µL | 552.7 (169.6-2951.0) | <50 vs. 201-500 cells/ µL | -98.98 | <0.001 | <0.001 |
| 201-500 cells/ µL | 951.0 (244.0-3715.0) | 51-200 vs. 201-500 cells/ µL | -26.03 | 0.34 | 0.12 |
| IL-8 cytokine levels by set CSF Leukocytes levels | | | | | |
| <50 cells/ µL | 300.1 (119.4-776.4) | <50 vs 51-200 cells/ µL | -70.82 | <0.001 | <0.001 |
| 51-200 cells/ µL | 827.1 (347.9-2104.0) | <50 vs. 201-500 cells/ µL | -83.48 | <0.001 | <0.001 |
| 201-500 cells/ µL | 912.4 (388.4-2158.0) | 51-200 vs. 201-500 cells/ µL | -12.66 | 0.64 | 0.23 |
| IL-13 cytokine levels by set CSF Leukocytes levels | | | | | |
| <50 cells/ µL | 21.8 (8.5-33.3) | <50 vs 51-200 cells/ µL | -57.47 | <0.001 | <0.001 |
| 51-200 cells/ µL | 33.3 (16.1-42.1) | <50 vs. 201-500 cells/ µL | -51.2 | 0.03 | 0.02 |
| 201-500 cells/ µL | 27.7 (20.2-37.7) | 51-200 vs. 201-500 cells/ µL | 6.271 | 0.82 | 0.29 |
| CXCL10/IP-10 chemokine levels by set CSF Leukocytes levels | | | | | |
| <50 cells/ µL | 2273 (842.2-2998.0) | <50 vs 51-200 cells/ µL | -54.38 | 0.001 | 0.001 |
| 51-200 cells/ µL | 2677.0 (2227.0-3226.0) | <50 vs. 201-500 cells/ µL | -60.09 | 0.01 | 0.006 |
| 201-500 cells/ µL | 2608.0 (2383.0-3037.0) | 51-200 vs. 201-500 cells/ µL | -5.707 | 0.84 | 0.29 |
| CCL11/Eotaxin chemokine levels by set CSF Leukocytes levels | | | | | |
| <50 cells/ µL | 13.6 (6.8-19.2) | <50 vs 51-200 cells/ µL | -65.12 | <0.001 | <0.001 |
| 51-200 cells/ µL | 19.9 (12.3-24.6) | <50 vs. 201-500 cells/ µL | -77.1 | 0.001 | <0.001 |
| 201-500 cells/ µL | 20.5 (14.2-23.1) | 51-200 vs. 201-500 cells/ µL | -11.98 | 0.66 | 0.23 |
| PD-L1 immune checkpoint levels by set CSF Leukocytes levels | | | | | |
| <50 cells/ µL | 86.2 (44.7-160.1) | <50 vs 51-200 cells/ µL | -70.17 | <0.001 | <0.001 |
| 51-200 cells/ µL | 167.2 (86.1-260.0) | <50 vs. 201-500 cells/ µL | -98.08 | <0.001 | <0.001 |
| 201-500 cells/ µL | 179.1 (122.2-287.6) | 51-200 vs. 201-500 cells/ µL | -27.92 | 0.31 | 0.11 |
| IL-10 cytokine levels by set CSF Leukocytes levels | | | | | |
| <50 cells/ µL | 209.6 (139.7-302.3) | <50 vs 51-200 cells/ µL | -80.56 | <0.001 | <0.001 |
| 51-200 cells/ µL | 299.4 (224.4-492.1) | <50 vs. 201-500 cells/ µL | -100.3 | <0.001 | <0.001 |
| 201-500 cells/ µL | 360.9 (257.8-406.3) | 51-200 vs. 201-500 cells/ µL | -19.76 | 0.47 | 0.16 |

Summary statistics from the two-stage linear step-up adjustment for multiple colinear differences in variable by set CSF leukocyte count using Benjamini, Krieger and Yekutieli model analysis. Unadjusted p Value <0.05 was consistently significant. The adjusted q value <0.05 was consistently significant.
