# Supplementary material for: Impact of cerebrospinal fluid leukocyte infiltration and activated neuroimmune mediators on survival with HIV-associated cryptococcal meningitis
Source: PLoS Negl Trop Dis. 2025 Feb 10;19(2):e0012873. doi: 10.1371/journal.pntd.0012873 (PMC11844869; doi:10.1371/journal.pntd.0012873)
Supplement: S3 Table — 1- Survival by 5 levels of CSF white blood cells. 2- Survival by 3 levels of CSF white blood cells. 3- Survival by 2 levels of white blood cells. (DOCX) [file pntd.0012873.s005.docx]

**Supplementary material S3 Table**. **The proportion of survival and the differences in survival curves by set levels of CSF white blood cells.**

1. **Survival by 5 white blood cell set categories**

| Survival data summary | Survival weeks; <5 CSF WBCs | Survival weeks; 5-20 CSF WBCs | Survival weeks; 21-50 CSF WBCs | Survival weeks; 51-100 CSF WBCs | Survival weeks; 101-200 CSF WBCs | Survival weeks; 201-500 CSF WBCs |
| --- | --- | --- | --- | --- | --- | --- |
| Number of rows | 400 | 400 | 400 | 400 | 400 | 400 |
| # of blank rows | 174 | 375 | 359 | 376 | 372 | 375 |
| # Rows with impossible data | 0 | 0 | 0 | 0 | 0 | 0 |
| # Censored subjects | 113 | 15 | 23 | 17 | 18 | 17 |
| # Deaths/events | 113 | 10 | 18 | 7 | 10 | 8 |
|  |  |  |  |  |  |  |
|  |  |  |  |  |  |  |

| **Comparison of Survival Curves** |  |
| --- | --- |
|  |  |
| Log-rank (Mantel-Cox) test (recommended) |  |
| Chi square | 8.179 |
| df | 5 |
| P value | 0.1466 |
| P value summary | ns |
| Are the survival curves sig different? | No |
|  |  |

CSF – cerebrospinal fluid

WBC – White blood cells

Ns -not statistically significant

1. **Survival by 3 white blood cell set categories**

| Survival data summary | Survival weeks; < 50 CSF WBCs | Survival weeks; 50-200 CSF WBCs | Survival­ weeks; 200-500 CSF WBCs |
| --- | --- | --- | --- |
| Number of rows | 400 | 400 | 400 |
| # of blank rows | 108 | 348 | 375 |
| # Rows with impossible data | 0 | 0 | 0 |
| # Censored subjects | 151 | 35 | 17 |
| # Ceaths/events | 141 | 17 | 8 |

| **Comparison of Survival Curves** |  |
| --- | --- |
|  |  |
| Log-rank (Mantel-Cox) test (recommended) |  |
| Chi square | 7.161 |
| df | 2 |
| P value | 0.0279 |
| P value summary | * |
| Are the survival curves sig different? | Yes |
|  |  |

CSF – cerebrospinal fluid

WBC – white blood cells

1. **Survival by 2 white blood cell set categories**

| Survival data summary | Survival weeks; =50 CSF WBCs | Survival weeks; >50 CSF WBCs |
| --- | --- | --- |
| Number of rows | 400 | 400 |
| # of blank rows | 108 | 323 |
| # rows with impossible data | 0 | 0 |
| # censored subjects | 151 | 52 |
| # deaths/events | 141 | 25 |

| **Comparison of Survival Curves** |  |  |
| --- | --- | --- |
|  |  |  |
| Log-rank (Mantel-Cox) test |  |  |
| Chi square | 7.144 |  |
| df | 1 |  |
| P value | 0.0075 |  |
| P value summary | ** |  |
| Are the survival curves sig different? | Yes |  |
|  |  |  |

CSF - Cerebrospinal fluid

WBC – white blood cells
