# Supplementary material for: Impact of cerebrospinal fluid leukocyte infiltration and activated neuroimmune mediators on survival with HIV-associated cryptococcal meningitis
Source: PLoS Negl Trop Dis. 2025 Feb 10;19(2):e0012873. doi: 10.1371/journal.pntd.0012873 (PMC11844869; doi:10.1371/journal.pntd.0012873)
Supplement: Attachment — Submitted filename: Responses_to_Peer_Reviewers_PNTD-D-24-00912..pdf [file pntd.0012873.s011.pdf]

**TO THE EDITOR/PEER REVIEWERS**
**PLOS NEGLECTED TROPICAL DISEASES**
**TRANSFERRED PEER REVIEWERS COMMENTS/QUERIES AND AUTHORS RESPONSES**

Dear Dr. Okurut:

Thank you very much for submitting your manuscript "Impact of Cerebrospinal Fluid Leukocyte Infiltration and Neuroimmmune Mediators on Survival with HIV-Associated Cryptococcal Meningitis" (PPATHOGENS-D-24-01093) for review by PLOS Pathogens.

As with all papers reviewed by the journal, your manuscript was reviewed by members of the editorial board and by several independent reviewers. Based on the reviews, we regret that we will not be pursuing this manuscript for publication at PLOS Pathogens.

The reviewers commented that the paper found some new interesting clinical correlations and complemented the high quality of work and clarity of presentation. However, they also indicated that the paper is incremental, especially in the context of work published recently by this group using the same cohort of patients. Considering this, we suggest the transfer (along with the reviews) to the PLOS Neglected Tropical Diseases, which would be a great fit for this scope of work upon some minor revisions.

The reviews are attached below this email, and we hope you will find them helpful if you decide to revise the manuscript for submission elsewhere.

While we cannot consider your manuscript further for publication in PLOS Pathogens, we would like to offer you the option to transfer your submission, with reviews, to PLOS Neglected Tropical Diseases https://www.editorialmanager.com/PNTD/

If you DO wish to transfer your submission, please click this link:

********

If you do NOT wish to transfer your submission, please click this link to decline:

********

Please note, all PLOS journals are editorially independent and vary in submission requirements.

Should you choose to transfer, your manuscript files, along with the reviewers' comments and their identities will be transferred automatically, and you will receive a confirmation email within 24 hours. Once transferred, your submission will be returned to you so you can check over your record before completing the submission. You may be asked to provide additional information, such as a response to the reviewers' comments. If you have any questions, please contact the editorial office of PLOS Neglected Tropical Diseases https://www.editorialmanager.com/PNTD/

We are sorry that the news is not more positive on this occasion, and we hope you will consider PLOS Pathogens for future submissions. Thank you for your support of PLOS and of open-access publishing.

Sincerely,

Michal Olszewski
Section Editor
PLOS Pathogens

Michael Malim
Editor-in-Chief
PLOS Pathogens
orcid.org/0000-0002-7699-2064

***********************

Reviewer's Responses to Questions

Reviewer's Responses to Questions

**Part I - Summary**
Please use this section to discuss strengths/weaknesses of study, novelty/significance, general execution and scholarship.

**Reviewer #1:** This study continues the analysis of CSF cytokines on specimens collected in 2015-2017. Four hundred one patients coinfected with HIV and Cryptococcus neoformans were studied at that time. A prior analysis of the same data (ref. 18) focused on gender differences, whereas this analysis focused on CSF leukocyte count. The current analysis confirms the prior studies showing that CSF leukocyte count, IL2, CXCL-10 and PD-L1 correlate with 18 week mortality (ref, 18, 34 and Jarvis, JID 2013; 207). The contribution of the current study is to show that low CSF leukocyte counts correlate with higher number of cryptococci, lower numbers of CD4 and CD8 cells , lower protein concentrations, and lower IL17A and TNF-alpha concentrations in CSF.

**Reviewers comment:** Limitations of the study are absence of data on follow-up, such as during IRIS, and no comparison data on blood cytokines.

**Authors response: (**a) The albescence of data on follow-up is a scope limitation. Our hypothesis was to defined outcomes based on baseline findings to be able to support and inform modality to improve outcome at diagnosis. This is important since majority of mortality outcome is reported during month of diagnosis with cryptococcal meningitis.
(b). The unmasking IRIS starts to show up with ART initial that is deferred 4-6 weeks later after diagnosis. Paradoxical IRIS is shown to start occurring at 10 weeks onwards. At this time of unmasking and paradoxical IRIS onset majority of the ensuing deaths after diagnosis would have occurred with survival curves flattening after 6 weeks. However, some of the later deaths can be attributed to IRIS episodes and correlates to these deaths is an area of future research.
(c). The analysis focused on CSF as a proxy for fungal activity in the CNS where fatal infection/disease manifests. We agree that, there might be difference in immune response between CSF and blood, but out earlier reports (Meya DB et al., 2015 and Okurut et al., 2020 showed compartmentalization of immune responses activated in CSF and blood. Moreover, these for not reported by function and by survival.

**Reviewer's comment:** The study was done at a time when mortality of cryptococcosis was much higher in Uganda (45%) than at the present. Correlates of mortality may have changed. Although 51% of the 401 patients were already receiving ART, no analysis of the possible impact prior ART on initial cytokines was done, probably assuming no IRIS was occuring.

**Authors response:** The reviewer brings out to perspective important facts about difference in mortality at the time the parent study was conducted and recent improved survival with cryptococcal meningitis. Okwir M et al., 2024 and Tenforde WM et al., 2020 shows that the improved survival is only experiences in high income research settings and at tertiary hospitals that are heavily involved in research. The sub analysis in earlier publication shown no major difference in immune response with ART experience (Okurut et al., 2023 (reference 18). The IRIS hypothesis would be a factor if most mortality was occurring after initial hospital discharge. Therefore, from the epidemiology point of point of view, though survival seems to improve overtime, the factors associated with an set of cryptococcal meningitis at diagnosis and correlates of survival may not have significantly changed.

**Reviewer's comment:** Considering that their prior analysis of this data found the largest cytokine differences to be in women, the reader is left to wonder whether many of the findings in this report reflect the findings in the 160 females and less so in the 241 males. It would be helpful if the authors more clearly distinguished what is different in this analysis from that in reference 18. They may also wish to point out that no adjustment was made for multiple comparisons, which impact the reader's interpretation of the p values in the 0.02-0.04 range in tables 2 and 3.

**Authors response: (a).** There is a clear distinction between the current findings and earlier report. The current findings are generalizable to cryptococcal meningitis immune response, fungal burden and survival outcome by CSF white cell counts. The earlier report is generalizable to cryptococcal meningitis neuroimmune response and survival by sex.
(b). The p values on Tables 2 and 3 show adjusted variables in each model.

**Reviewer's comment:** The writing is clear and tables useful. The figures are also useful but figure 3 had three different graphs, a, b and c, when only one (a) tells the entire story. The graphs in D of that figure include two univariate analyses that didn't hold up in multivariate analyses, questioning their worth.

**Authors response: (a).** Thank you acknowledging the clarity and usefulness the data on Tables and Figures. (b). The approach taken for Figure 3 data illustration was purposely to benefit diversity of readership.

**Reviewer's comment:** A few comments on wording are as follows:
Lines 131-136 have a list separated by periods that should be separated by semicolons, not listed as separate, incomplete sentences.

**Authors response:** Revised. Refer to line 130.

**Reviewer's comment:** Lines 143-144 more details are needed to explain how missing survival data were imputed. Their prior analysis (ref. 18) did not include imputed data.

**Authors response:** Revised. Refer to line 144-145.

**Reviewer #2:** In this study, Okurut et al. examine CSF profiles in 401 patients with HIV-associated cryptococcal meningitis (CM) in three hospitals in Uganda. The stated purpose of the study was to investigate the association of CSF leukocyte counts and immune responses plus survival. Patients were stratified based on their CSF WBC counts into three groups: ≤50/µL, 51-200 cells/µL, and 201-500 cells/µL. The CSF samples, which were originally collected as part of a study examining the effect of adjuvant sertraline for the treatment of CM, were then examined for chemokine and cytokine levels and other selected markers by Luminex.

Strengths of the study include strong investigative teams working in Uganda and the United States. The authors have navigated many logistical hurdles necessary for sample collection, storage, and analysis. The studies are conducted well. The discussion is thoughtful. The findings have potential translational relevance. Some novel associations were uncovered which could form the basis for future hypotheses and studies.

**Authors response:** Thank for this interesting narrative and appreciation of our team's collaborative efforts.

**Reviewer's comment:** Weaknesses of the study include its exploratory, descriptive nature. There are no ex vivo functional correlations using the CSF samples. The authors have an outstanding track record of performing high impact clinical studies on CM in Uganda. A prospective validation phase using samples from a second clinical study would greatly increase the strength of the conclusions. An additional weakness is others have performed similar studies with similar correlations (such as correlation of survival with CSF WBC and CFUs; however, as noted above, there are novel correlations in the manuscript). It is not surprising to find that cytokine and chemokine levels correlate with WBC counts considering that most of the studied cytokines and chemokines are made by WBCs.

**Authors response: (a).** We would like to point out that though the current study is weak because of its exploratory and descriptive nature, it forms a strong foundation for future studies to dive deeper to defining underlying functional mechanisms to observed findings.
(b). Much as it is not surprising to find cytokine relationships with WBCs, the clear illustrations of facts especially in a population of patients where fatal diseases manifests with severe immune suppression is novel. In fact, with our findings, we can clearly propose adjunctive immune based booster therapies, because we now clearly know the population that could benefit from such therapies.
(c). Earlier others have demonstrated patching relationships of CSF WBCs and CFUs but, none of the reports ever demonstrated comprehensively the association of CSF WBCs, CFUs, immune response and survival. We are happy that the reviewer agrees that this was a novel approach taken by the authors.

**Reviewer #3:** I had the opportunity to review the manuscript by Okurut and colleagues. This is a well-written an interesting manuscript that was performed as a substudy of a clinical trial completed in 2018 among over 400 African patients with cryptococcal meningitis. This study focuses on CSF leukocyte response and associated measures of immunologic function as it relates to survival. Not surprisingly, patients with a more robust CSF white cell response (at least 50 cells per cubic millimeter) had a significantly better 18-week overall survival. This is not terribly surprising giving some the early observations from the first cryptococcal meningitis studies of Bennett and Dismukes published in the late 70s and 80s. These authors observed that having a CSF white count of less than 20 tended a correlate with poor outcome overall. Of course at the time there was very limited capacity to perform immunologic measures that are more feasible today. Nonetheless the findings from the current study are consistent with some of these observations in early trials which did not include HIV patients.

**Authors response: (a).** Thank for this interesting narrative and appreciation of our team's collaborative efforts in the current state of research with advancement in technology. We are happy to note that our findings relate to former observations and move forward the science.

**Reviewer's comment:** The text is easy to follow and the authors present logical arguments to further position and this is well buttressed with supportive data.

**Authors response:** Thank you.

**Reviewer's comment:** Again, this is not surprising given that interferon gamma, IL-17 and other related chemokines correlate with a more robust inflammatory response.

**Authors response:** The findings go beyond demonstrating the correlates of an inflammatory response to Cryptococcus to comprehensively defining immune compromised host at risk of deaths from rather a preventable cause that is potentially reversible by an adjunctive immune based therapy.

**Reviewer's comment:** I, do however, disagree with one of the authors' final conclusions suggesting that the treatment of cryptococcal meningitis is incomplete with antifungal therapy alone. Certainly this is not supported by clinical trial data. It may be more accurate to state that among a certain group of patients adjunctive immunotherapy may be helpful in both clearing the organism and improving overall survival. The problem of course is that not all patients need pro inflammatory support such as adjunctive interferon gamma, but rather might be benefit from tamping down the inflammatory response with an agent such as prednisone. I think it would be helpful to acknowledge the shortcomings of the current data, as it does not address the issue of adjunctive immunotherapy. Suggest simply resting on the fact that HIV patients with cryptococcal meningitis who have a relatively preserved leukocyte CSF response, and in whom an appropriate immunologic modulator response is present, appear to do better overall when compared to their counterparts who lack a more robust inflammatory response.

**Authors response:** We appreciate the reviewers mixed reaction to findings and the alternative approached to moving forward the results. These concerns have been duly acknowledged and text revised. Please refer to section on the conclusion on lines 338-343.

**Reviewer's comment:** Finally, I think the authors have done a good job of presenting the data clearly. I would just caution to not over interpret their findings as I have suggested above.

**Authors response:** Thank you too for taking the time to review our work. Thank you for an in-depth discussion and additions that have improved the study conclusions and projected the future utility of study results.

--------------------

**Part II – Major Issues: Key Experiments Required for Acceptance**
Please use this section to detail the key new experiments or modifications of existing experiments that should be <u>absolutely</u> required to validate study conclusions.

Generally, there should be no more than 3 such required experiments or major modifications for a "Major Revision" recommendation. If more than 3 experiments are necessary to validate the study conclusions, then you are encouraged to recommend "Reject".

**Reviewer #1**: no additional analysis is needed.
**Reviewer #2:** none
**Reviewer #3:** NA

**Authors response:** Thank you for the agreement among three independent reviewers and trust on our results.

--------------------

**Part III – Minor Issues: Editorial and Data Presentation Modifications**
Please use this section for editorial suggestions as well as relatively minor modifications of existing data that would enhance clarity.

Reviewer #1: see above

**Authors response:** All concerned raised by the reviewer were appropriately addressed.

**Reviewer #2:** 1. If the information is available, I suggest adding CSF CrAg titers to Table 1 or Figure 1.

**Authors response:** The CSF CrAg titers were not done for this particular cohort.

**Reviewer's comment:** 2. Figure 1A. It seems nonsensical to include statistics. Of course it will be significant, as the basis of stratification of the groups is the CSF WBC.

**Authors response:** The addition of statistics is to point to the readers what is relevant and clear doubt as to whether the observable differences were statistically significant. This approach of data description was in line with scientific method that was used to define important results.

**Reviewer's comment:** 3. The figure 1 and 2 legends should define what the asterisks denote; only * is shown in the legend yet the figure has **, ***, and ****.

**Authors response:** Revised. Refer to Figure 1 legend line 598 and Figure 2 legend line 614

**Reviewer's comment:** 4. Line 163. PLOS does not accept references to data not shown. The Pearson r value should be provided.

**Authors response:** Pearson r included. Please refer to line 165-166

**Reviewer's comment** 5. Line 277. I do not understand what the authors mean when they write "cryptococcal yeast differentiation".

**Reviewer's comment:** Revised. Please refer to line 280.

**Reviewer's comment** 6. Line 334. I disagree with this sentence as worded. Although relapse in the absence of immune reconstitution can be seen, many patients with CM appear to be cured by antifungal therapy.

**Authors response:** Revised: Please refer to lines 338-343.

Reviewer #3: NA

--------------------

PLOS authors have the option to publish the peer review history of their article (what does this mean?). If published, this will include your full peer review and any attached files.

If you choose "no", your identity will remain anonymous but your review may still be made public.

**Do you want your identity to be public for this peer review?** For information about this choice, including consent withdrawal, please see our Privacy Policy.

Reviewer #1: No

Reviewer #2: No

Reviewer #3: No
