# Supplementary material for: Impact of cerebrospinal fluid leukocyte infiltration and activated neuroimmune mediators on survival with HIV-associated cryptococcal meningitis
Source: PLoS Negl Trop Dis. 2025 Feb 10;19(2):e0012873. doi: 10.1371/journal.pntd.0012873 (PMC11844869; doi:10.1371/journal.pntd.0012873)
Supplement: Attachment — Submitted filename: Response to reviewers_PNTD-D-24-00912_12Oct2024.pdf [file pntd.0012873.s012.pdf]

Response to Reviewers, PLOS NTD

Manuscript ID: PNTD-D-24-00912R1

Manuscript title, "Impact of Cerebrospinal Fluid Leukocyte Infiltration and Activated Neuroimmune Mediators on Survival with HIV-Associated Cryptococcal Meningitis"

Date: 12th October 2024

Dear Editor,

Important note from authors to the Editor: Please find below the responses to the peer reviewers comment, edits and suggestions that guided the manuscript line for line revisions. Beyond what the peer reviewers had cited, and not included in here, the manuscript was revised based on the journal instructions to authors and based initial proofs from journal administrators review all highlighted in the revised version of the resubmitted manuscript. Additional revision included references formatting to Vancouver referencing style. Addition of the author summary, and supplementary materials.

Below is the list of line for line addressed revisions from journal peer reviewers comments, edits and suggested revisions.

[1] A letter containing a detailed list of your responses to the review comments and a description of the changes you have made in the manuscript. Please note while forming your response, if your article is accepted, you may have the opportunity to make the peer review history publicly available. The record will include editor decision letters (with reviews) and your responses to reviewer comments. If eligible, we will contact you to opt in or out.

[2] Two versions of the revised manuscript: one with either highlights or tracked changes denoting where the text has been changed; the other a clean version (uploaded as the manuscript file).

Important additional instructions are given below your reviewer comments.

Please prepare and submit your revised manuscript within 60 days. If you anticipate any delay, please let us know the expected resubmission date by replying to this email. Please note that revised manuscripts received after the 60-day due date may require evaluation and peer review similar to newly submitted manuscripts.

Reviewer #1: The present study expands knowledge about the immune response in the CNS in patients with cryptococcal meningitis. The authors focus on immune elements present in CSF. However, for greater completeness of their findings, they could include in their results the antigenic titer (CrAg titer), as well as the measurement of glucose in the CNS.

Authors Response: *Antigenic titers measurement would have been interesting to correlate with fungal burden and immune response. However, antigenic titers measurement was not done in the project.*

Reviewer #1: The conclusions of the manuscript have been extensively reviewed previously. Due to the fact that cryptococcal titers interfere with the host's immune response, it would be interesting to include these measures in the present work.

Authors Response: *The antigenic titers measurement was not done in the parent or the present study. The proxy to antigenic titers is the fungal. At baseline, cryptococcal fungal burden in CSF is directly proportional to the fungal titers. This relationship is only altered with antifungal treatment initiation. (Jonathan Dyal, Med Mycol. 2016 May 1; 54(4): 361–369. doi:10.1093/mmy/myv104) and Annemarie E et al., 20 JID 2005; 192:681–4).*
The study presents data at baseline, prior to antifungal treatment initiation. Based on the available evidence, there may not be a need to reinvent the will. The study findings can be extrapolated with high confidence to correlate with both fungal burden and fungal titers.

Reviewer #1: Furthermore, as they probably have CSF samples, they could quickly measure glucose, which is an important factor in poor prognosis in these patients.

Authors Response: *The CSF glucose was measured at baseline but did not differ statistically by CSF white cell counts. Table 1.*

Reviewer #2: The manuscript presents an analysis of selected data from the ASTRO trial exploring associations between CSF white cell count, CSF immune markers, and survival. The overall findings are as would be expected based on prior published literature, and there are some novel data on immune markers that have not previously been studies in detail. Better CSF white cell counts are associated with higher peripheral CD4 T-cell counts and more active CSF inflammation, which is in turn associated with lower fungal burden and survival. These corroborative findings are worth publishing and add to the literature. However, the current analysis is very difficult to follow and has several major methodological limitations that significantly weaken the paper. The interpretation of the findings often goes beyond what the data show. These issues are listed below under major comments, with some suggestions as to how they could be addressed.

Major comments

Reviewer 2: Q1 (a). A clear description of the study population should be given. Were they all participants in the ASTRO trial? If not, how were they selected. What treatment did they get?

Authors Response: *The issues raised by the reviewer in this section have been thoroughly revised and addressed on (Lines 98-119). Specifically, all participants were all from ASTRO trial. The participants were randomized to either adjunctive Sertraline (the trial arm) or to the standard WHO recommended antifungal therapy (Placebo arm). There was no survival benefit of the trial drug shown by no difference in survival by randomization.*

Reviewer 2: Q1 (b). Why is there missing outcome data if these were trial participants?

Authors Response: *Missing was reported because the trial operated in real-world setting with expected logistics limitations that are common in third world settings that affected communications. Though patients were enrolled during in-hospital admission, participants were follow-up for 18 weeks for survival outcomes. Lack of information on the where about or survival of patients after 3 consecutive given phone contacts was reported as missing.*

Reviewer 2: Q1 (c). How many had missing outcome data?

Authors Response: *Sample size is included for every sub analysis for the reported findings implied to respond to this question.*

Reviewer 2: Q1 (d). How and why was imputation for missing outcomes performed?

Authors Response: *The approach taken in the imputation is explained in every section where missingness in survival data was imputed Lines 170-182; 258-259; and 274-275.*

Reviewer 2: Q1 (e). And why not exclude those with missing outcomes from the survival analysis?

Authors Response: *All authors unanimously agreed to have survival sensitive analysis performed in order not to exclude anyone just because they were missing survival outcome data. As an approach taken in modern and advanced statistical analysis, given that missingness was minimal (<10% of the cohort).*
The reported results did not differ with survival outcome imputation (Lines 258-259 and Lines 274-275)

Reviewer 2: Q2 (a). How were the primary exposure and outcome variables selected?
In terms of CSF WCC categories, from the presented graphs, it appears that the authors split the data multiple ways then selected the categories that gave the results they needed. I presume that this was not the case, and that categories were based on biological plausible ranges. This should be clearly defined and stated in the methods. With the current categorization, the groups are very unbalanced in size. Almost everyone is in the <50 group (318) compared to just 26 in the higher group. This leads to a very large loss of power with most of the data spread in terms of WCC minimized into a single <50 category. Prior analyses have used <5 or <20 as cut points. It would seem to make much more sense to do the same here from any a priori knowledge perspective and the spread of the data. The only obvious reason to select the current categories is based on conditioning on the desired outcome (mortality) as seen in Figure 3A. From a statistical point of view this is a weak methodology and prone to bias.

Authors Response: *Revised (Lines 126-150). Yes, we took a different unique approach in this study that informed new biological plausible priori range for future investigations in HIV associated cryptococcal meningitis immune response, fungal burden and survival associated investigation. The initial data stratification was based on small priori ranges as stated by the reviewer. This led to result on (mortality) in Figure 3A, that trickled down to Figure 3B-C. Based on this mortality outcome figure we worked backwards to interrogated to the data to examine if patients in this data category had unique attributes to fungal burden and immune response at baseline. And yes, by the chosen patients' white blood cell clusters, the patients are distinct by survival outcomes attributes, fungal burden and evoked immune response. This informs new and unique biological plausible ranges for future priori investigations based fungal burden, CSF white blood cells, immune response and survival with antifungal therapy.*

Reviewer 2: Q2, (b). In terms of mortality, why was 18 week survival chosen? Cryptococcal specific mortality, rather than that due to profound immune suppression and other OIs, is likely better captured at earlier time points. Were data similar for 2 or 10 week mortality?

Authors Response: *Revised lines 128. The overall 18 week survival was the trial primary endpoint. Survival overall, started to differ after only 3 weeks and by 6 week the curves had started to flatten (Figure 3 A-C).*

Reviewer 2: Q3, (b). The current analysis has a problem with multiple comparisons, and no adjustment for multiplicity in the analysis. The authors do not state how many pairwise comparisons were made, but it is clearly a considerable number. A further problem with these type of datasets is that very few of the variables are independent of each other, and likely to be very closely correlated, all really being markers of the same underlying immune response. It is therefore very difficult to use lots of individual associations or multivariable analyses using regression models to draw robust statistical conclusions. The raw data show a very clear story here, and as presented, the analysis over complicates things and makes the central message less clear. The authors could adjust for multiple comparisons. However, this may not be the best methodology here. It may be best to just clearly present the raw data as has been done to some extent in Figures 2 and 3.

Authors Response: *adjustment for multiple comparison does not alter the outcome. Hence there is not better way to represent the results compared to what the authors did. Refer to the Supplementary Table S1-S2.*

Reviewer 2. A different PCA could be performed to reduce the dimensionality of all of the combined the CSF immune markers, and then see what contributes most to PC1, PC2, etc. and how these correlates with the CSF WCC and other key variables, and outcomes.

Authors Response: *Another truncated PCA summary of only cytokine and chemokine data has been added. Yes, it helps to improve clarity of the data and data interpretation. Figure 4 C.*

Reviewer QN 4. The current PCA is not well described and very hard to interpret. What was added into the PC analysis? How were the components derived? What were the weightings of the individual components? How did the components differ by CSF WCC category? By mortality? Furthermore, it is stated that adjustments were then made for PC scores in other analyses. As these PC scores were presumably derived from and correlated with the parameters being included in these analyses this does not make sense. Why do this and what does it mean? The PCA plots are also very hard to interpret. Survival has been included, but is also what is being predicted in subsequent analyses using the PCA. The plot showing proportion of variance would usually show this for PC1, PC2, etc, and be used to guide which components to use.

Authors response: *The PCA analysis and data description has been revised in detail. Please refer to Figure 4 and lines 249 – 379.*

Reviewer QN 5. The descriptions of key results in the results section are difficult to follow. For example, Line 168 "CSF cytokines and chemokine concentration positively correlate with CSF leukocytes infiltration". Presumably the association is with the CSF WCC categories that have been set? If so, state this. Or is it as a continuous variable? And what measures of correlation were used?

Authors Response: *this result was revised. Please refer to lines 222-223*

Reviewer QN 6. Likewise, when describing survival associations in Line 188, what measures of association were used? Derived from which analyses? The raw data are clear here, so perhaps just present visually and describe.

Authors Response: *this result was revised. Please refer to lines 237*

Reviewer QN 7. "Table 2 Model 1: (PC1; (ii)) CSF white cell dependent and matched independent colinear predictor variables. Adjusted for PCA cluster." This is very hard to interpret, both in terms of methodology used, and what the results mean. In terms of analysis, wasn't the PCA cluser derived from the predictor variables in the first place? And how can the results be meaningfully interpreted? What is a -8 fold risk with each increase in log10 CFU? In what? This applies to all other variables in the table.

Authors response: *The interpretation of this results depends on an understanding if the PCA model. This results description has been merged to the PCA results description and interpretation to guide the readers and ease understanding. Please refer to Figure 4 and lines 249 – 379.*

8. Table 3 – why present both linear and logistic regressions? How were these models constructed? What do these risk ratios and odds ratios mean? It is very hard to interpret the numbers.

Authors response: *The interpretation of this results depends on an understanding if the PCA model. This results description has been merged to the PCA results description and interpretation to guide the readers and ease understanding. Please refer to Figure 4 and lines 249 – 379.*

Reviewer QN 9. Figure 1(A) shows a statistically significant difference in CSF WCC in the CSF WCC categories that have been constructed by the authors. This is an inappropriate use of statistical inference and should be removed. Throughout Figs 1 and 2 the statistical testing takes no account of multiplicity. This should either be corrected for of a different statistical approach taken – or a justification given for why no multiplicity adjustments would be needed.

Authors Response: *The figure 1-2 footnotes have been revised. Please refer to line 888-895 and lines 963-973.*

Reviewer 2 QN 10. Fig 3 – Part (D) is hard to reconcile with text. What are the units? It looks like there is no difference in CSF IFNg between those who lived and died, no difference in protein, no difference in PD1, and no difference in IL2. This is not what is reported in text or conclusions.

Authors Response: *Revised line 1019- Figure 3 D, responses mostly the observed differences in in immune response between survivors and patients who died during the 18 weeks of follow up.*

Reviewer 2. QN11. In the discussion, Line 268, it is stated that "In this regard, the immune damage response framework suggests that antifungal therapy alone without immune modulatory therapy is not sufficient to cure the infection and modulate host immune

mediated injury and death." This is not true, and in no way supported by the data presented. With modern antifungal therapy the majority of individuals with cryptococcal meningitis will be cured.

<u>Authors Response</u>: *Lines 414-415. Revised. Thank you.*

Reviewer 2, QN12. Other conclusions are too far reaching and not based on the data presented. For example, in Line 274 "The inverse association of the CSF fungal burden with the levels of cellular and soluble immune response and host survival implicates the selective evasion of Th1- and Th17-mediate[d] control of intracellular Cryptococcus replication."

<u>Authors Response</u>: *Revised – Lines 438-440.*

Reviewer 2, QN And Line 299 "These observations are consistent with a T cell evasion hypothesis". Neither of these are likely to be true. There is not selective evasion of Th1 and Th17 responses or T-cell immunity – there is just a profound lack of T-cell mediated immunity overall in a population of individuals with advanced HIV disease and very low CD4 counts. If you have more CD4 cells you have more CSF immune activation and better outcomes.
<u>Authors Response</u>: *Revised line 463*

Reviewer 2, QN13. Further lines in the discussion could be reworded for clarity. It is hard to determine the meaning of the following sentences:
Line 330 "The decrement in these factors likely conspire to limit T cell-mediated control of intracellular cryptococcal replication in macrophages with resultant serious infection and death". And Line 339 "This exempts survival improvement tapering therapy for exacerbated inflammatory response with possible persistently elevated intracranial pressure and immune reconstitution inflammatory syndrome."

<u>Authors Response</u>: *The conclusion has been extensive revised to contextualize and clearly bring out the key findings of the study. Please refer to lines 501-512*

<u>Minor comments paucity</u>
Reviewer 2 QN14. Line 66. "Monocytes, the precursors of macrophages that harbor intracellular replication machinery for Cryptococcus". This is not really correct. Cryptococci can replicate inside monocyte lineage cells, but they don't "harbor replication machinery".

<u>Authors Response</u>: *Line 66; Revised. Thank you.*

Reviewer 2 QN15. Line 75. "The immune-activated cytokine, chemokine, and checkpoint regulatory responses are important in orchestrating sequestration of Cryptococci in the CNS". What does this mean? Is sequestration the correct term here?

<u>Authors Response</u>: *Line 75-76; Revised. Thank you.*

Reviewer 2 QN 16. Line 165. "The peripheral white blood cells only had a trend to increasing with CSF white cell stratification (Figure 1 D)." A trend is a specific statistical term, and does not mean a p-value of somewhere near 0.05. Say there was no significant association.

Authors Response: *Lines 209-210. Revised. Thank you.*

Reviewer #3: The authors reported the associations between Cerebrospinal Fluid Leukocyte Infiltration and Neuroimmmune Mediators in HIV-Associated Cryptococcal Meningitis. Although the study provide some new insights to research area, there are some severe deficiencies in the present study:

1) the chemokines/cytokines in CSF are associated with leukocyte count and fungal burden in CSF. It is recognized that fungal burden and Neuroimmmune Mediators are potentially associated with CSF open pressure, which is a determinant of mortality of patients. the present study did not reveal the relationships between Neuroimmmune Mediators and CSF open pressure. Specially, the CSF open pressure was not included in multivariate analysis, which would produce severe bias.

Authors Response*: Table 1, CSF opening pressure did not differ by CSF leukocytes set categories. Hence, there was no further concern for further investigations on the differences and confounding based on CSF opening pressure. If any, it has little impact on clearly unique and delineated and prooven results.*

2) How many patients accepted VP shunt? were there all participants did not adapt management to control CSF pressure? the effect of medical controlling of CSF on mortality is not reflected in statistics.

Authors Response*: The control of CSF opening pressure is a standard of care for cryptococcal meningitis patients. Every patient is managed with serial therapeutic lumbar puncture of the opening pressures clinically indicate so.*

3) the author declared that there were 51.5% (206/400) were antiretroviral therapy (ART) experienced. So, the cytokine/chemokine concentration is probably associated with IRIS. IRIS is profoundly affecting the profile of CSF Neuroimmmune Mediators. Consequently, the mixed ART experienced and ART naive patients is not convinced on statistics.

Authors Response*: No! all cytokines/chemokine measurement were performed at baseline at the time of meningitis diagnosis. IRIS in this patient according to the literature occurs at least after 10 weeks after cryptococcal meningitis diagnosis. The earlier Okurut et al., report of divergent neuroimmune response by sex, did not find difference by ART exposure.*

Figure Files:
While revising your submission, please upload your figure files to the Preflight Analysis and Conversion Engine (PACE) digital diagnostic tool, https://pacev2.apexcovantage.com. PACE helps ensure that figures meet PLOS requirements. To use PACE, you must first register as a user. Then, login and navigate to the UPLOAD tab, where you will find detailed instructions on how to use the tool. If you encounter any issues or have any questions when using PACE, please email us at figures@plos.org.

Authors Response*: Correct*

Data Requirements:

Please note that, as a condition of publication, PLOS' data policy requires that you make available all data used to draw the conclusions outlined in your manuscript. Data must be deposited in an appropriate repository, included within the body of the manuscript, or uploaded as supporting information. This includes all numerical values that were used to generate graphs, histograms etc.. For an example see here: http://www.plosbiology.org/article/info%3Adoi%2F10.1371%2Fjournal.pbio.1001908#s5.

Authors Response*: All the data used to draw conclusion of extensively presented and cited in the manuscript.*

Reproducibility: To enhance the reproducibility of your results, we recommend that you deposit your laboratory protocols in protocols.io, where a protocol can be assigned its own identifier (DOI) such that it can be cited independently in the future. Additionally, PLOS ONE offers an option to publish peer-reviewed clinical study protocols. Read more information on sharing protocols at https://plos.org/protocols?utm_medium=editorial-email&utm_source=authorletters&utm_campaign=protocols

Authors Response*: The protocol used to generate the data is was revised and is cited and referenced in the manuscript.*
