# Supplementary material for: Impact of cerebrospinal fluid leukocyte infiltration and activated neuroimmune mediators on survival with HIV-associated cryptococcal meningitis
Source: PLoS Negl Trop Dis. 2025 Feb 10;19(2):e0012873. doi: 10.1371/journal.pntd.0012873 (PMC11844869; doi:10.1371/journal.pntd.0012873)
Supplement: Attachment — Submitted filename: Okurut S_PNTD-D-24-00912R2_Responses to reviewer_17Jan25.docx [file pntd.0012873.s013.docx]

**Responses to reviewer**

**Comment 1**. Clarification was requested about selection of trial participants. It is stated in the response that they were all ASTRO participants. However, not all ASTRO participants are included here; there are 401 participants in this analysis and 460 in the trial. How were these 401 selected? Why not include all ASTRO participants? Please specify in the methods.

Response 1. All participants with available stored CSF were included. We have reworded in lines 115-121.

“(Why did 13% not have CSF available? A variety of reasons: sample volume, weekends/holidays, lab error. These samples are missing at random.)

**Comment 2**. If adjustment for multiple comparisons made no difference to the outcomes, then present all of the results correctly adjusted for multiple comparisons.

Response 2. Revised. The results are presented on Table 2-3 as regression coefficients

**Comment 3.** The following wording at line 81 is perhaps not accurately reflective of the pathology of cryptococcal disease in this population: "suggest an influence of cryptococcal and/or other underlying immune suppressive factors that selectively evade T helper (Th) - mediated defense. The evasion of the T cell helper function allows unchecked intracellular fungal replication leading to high fungal burden among patients and a lower probability of recovery." I don't think it is evasion of a functional T-cell response that is the problem here - rather it is a lack of a functional T-cell response due to the fact that these individuals have very advanced HIV disease. Could this be reworded?

Response 3: we have re-worded this section for clarity; Lines 83-88.

**Comment 4**. In the results, the wording at line 216 is not clear: "This association of CSF WBC number and survival was consistent with fewer deaths (Fig 3C) and more WBC strata (Fig 3C) (stratified adjusted Hazard Ratio, (HR= 1.634, 95% 218 CI; 1.140 to 2.343) and p=0.008) (Fig 3C) and supporting materials (S3 Table)." Please reword for clarity.

Response 4 A. We have re-worded this section for clarity; lines 233-235.

**Comment 4 B**: Line 218 “CSF white cell count was positively associated with better survival (Fig 3C). This association was consistent as a continuous factor as well across CSF white cell strata (stratified adjusted Hazard Ratio = 1.634, 95%CI; 1.140 to 2.343; p=0.008)."

Response 4B: This statement was reworded; lines 233-235

**Comment 5**. The same comment for line 223: "These findings were statistically consisted mostly for survival with the set three white blood cells categories except for high CSF fungal burden and low levels of CXCL10 that differentiated those who died with ≤50 CSF white cells/μL from survivors (S2 Fig)." Please reword for clarity.

Response 5. This confusing statement was deleted; lines 240-242

**Comment 6.** The description of the PCA remains very hard to follow. Several statements are factually incorrect. For example:
"Principal component analysis shows independent principal component clustering". This is not correct - principal components are by definition NOT clustered with each other. This is how they are derived.

Response 6A. This statement was deleted and subsequent section rewarded for PCA description; line 249-260.

**Comment 6B:** And "The principal component with highest effect sizes are those with the highest likelihood to predict model outcome" again is not correct; principal components do not have an intrinsic effect size - they simply reflect where there is variance within a dataset. Further rewording for clarity could help with interpretation.

Response 6B: We have reworded this as: “The principal component with highest effect sizes are those that contribute the highest variance to the model outcome”. We also have reworded the PCA results section; line 247-305.

**Comment 7**. In the discussion and conclusion, a few statements are either difficult to interpret, or over complicate the presented data. For example:

"Herein we describe a potential role of a combined polyfunctional immune elevated modulated response with influence to lower fungal burden and improve survival". What is a "combined polyfunctional immune elevated modulated response"? Could this be described more simply in a way that directly reflects the findings presented? In what way is the response "modulated"?

Response 7A. We have modified the text to make our broader point: line 344-348; “Herein we describe the dysfunctional response to cryptococcal infection with advanced HIV disease with dysfunctional CD4 T cells. Yet not all dysfunctional responses are created equal, and certain compensatory immune responses are associated with lower fungal burden and improved survival.”

**Comment 7b**. "Multiple host cellular and activated CSF soluble immune mediated elements appear to regulate adaptive-innate immune crosstalk to regulate cryptococcal fungal burden and host recovery." I don't see anything in these data supporting regulation of adaptive innate crosstalk, or how this regulation is impacting host recovery. There are very clear findings to take away from this work without the need to speculate or overcomplicate.

Response 7b. We have deleted this speculation and reworded the sentence as: lines 411-413; Patients with cryptococcal meningitis who have elevated levels of CSF soluble cytokines, chemokine and immune checkpoint elements, CSF leukocytes and circulating CD4^+^ and CD8^+^ T cell responses have lower fungal burden and high probability of survival.

**Comment 8.** In Figure 1 A(i) please remove statistical testing of pre-defined white cell categories as previously requested. The bars showing medians can be presented, but it is methodologically incorrect to perform statistical testing and draw inferences about the probability of there being a true difference between these groups when you have created these groups to be different. The probability that they are different is 100% as you have made them different.

Response 8. As groups are made by grouping WBCs, we concur, p values should not be presented and are removed; Fig 1.

**Comment 9**. Table 2. Although explanation has been added, these risk ratios are very hard to interpret. How can you have a negative risk ratio? A risk ratio of 1 indicates no difference. A risk ratio above 1 indicates and increased risk. A risk ratio below 1 indicates reduced risk (e.g. RR 0.5 would indicate a 50% lower risk) What does an RR of -8 mean? Are you presenting risk ratios here or something else? A coefficient from a regression model perhaps?

Response 9: We now represent these comparisons as regression coefficients; table 2.

**Comment 10**. Both risk ratios and odds ratios are still presented in the next table (with the same caveats about -ve numbers for the RRs as above) - why use both? Depending on the analysis, stick to one or the other.

Response 10: We now represent these comparisons as regression coefficients; table 3.
